# Supplementary material for: Impact of Drying Methods on Phenolic Components and Antioxidant Activity of Sea Buckthorn (Hippophae rhamnoides L.) Berries from Different Varieties in China
Source: Molecules. 2021 Nov 26;26(23):7189. doi: 10.3390/molecules26237189 (PMC8659002; doi:10.3390/molecules26237189)
Supplement: Supplementary file 1 [file molecules-26-07189-s001.zip › molecules-1478119-supplementary.pdf]

Supplementary Materials

Impact of Drying Methods on Phenolic Components and Antioxidant Activity of Sea Buckthorn (*Hippophae Rhamnoides* L.) Berries from Different Varieties in China

Yue Li [1,2], Pei Li [1,2], Kai-Lin Yang [1,2], Qian He [1,3], Yue Wang [1,2], Yu-Hua Sun [4], Chun-Nian He [1,2,4,*] and Pei-Gen Xiao [1,2]

[1] Institute of Medicinal Plant Development, Chinese Academy of Medicinal Sciences & Peking Union Medicinal College, Beijing 100193, China; 18073049745@163.com (Y.L.); lee_p1214@163.com (P.L.); yangkailin199908@163.com (K.-L.Y.); heqian971003@163.com (Q.H.); wywxc1019@163.com (Y.W.); pgxiao@implad.ac.cn (P.-G.X)

[2] Key Laboratory of Bioactive Substances and Resources Utilisation of Chinese Herbal Medicine, Ministry of Education, Beijing 100193, China

[3] Baotou Medical College, Baotao 014060, China

[4] Xinjiang Key Laboratory for Uighur Medicines, Xinjiang Institute of Materia Medica, Urumqi, 830004, China; sunyuh1117@aliyum.com

* Correspondence: cnhe@implad.ac.cn

Figure S1. UPLC-DAD chromatogram of mixed standard solution at 254nm (A) and W1 sample at 360nm (B).

Figure S2. Antioxidant Potency Composite (APC) index of sea buckthorn.

Table S1. The longitudinal diameter (mm) of sea buckthorn berries.

Table S2. The validation results of UPLC-DAD method: (A) Regression equation, LOQ and LOD of the twelve analyzed compounds. (B) Recovery rates for the twelve analyzed components.

Table S3. The content of 12 flavonoids and phenolic acids in sea buckthorn berries of different varieties and drying methods.

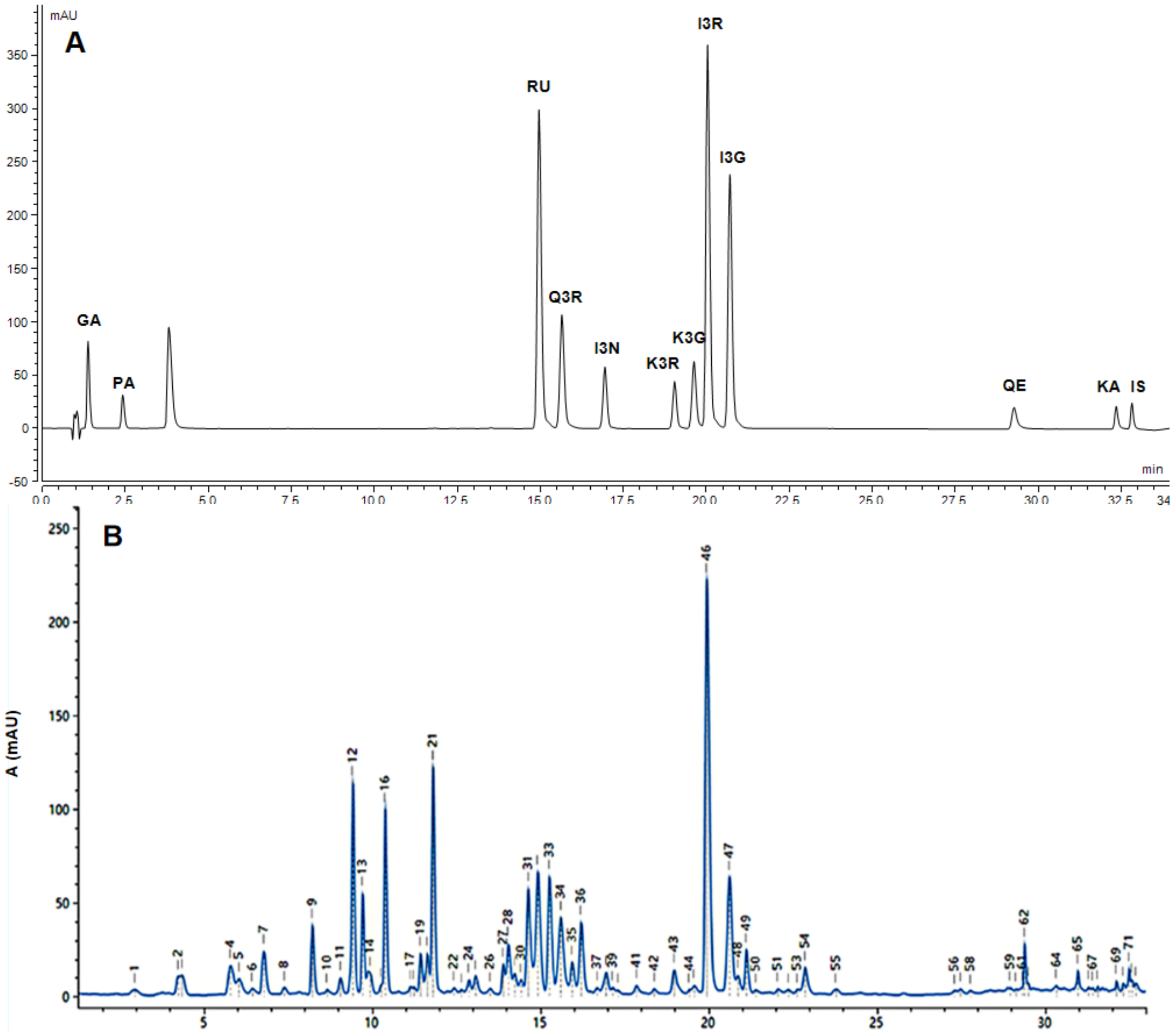

Figure S1. UPLC-DAD chromatogram: (A) mixed standard solutions at 254nm; (B) W1 sample at 360nm; peaks between 1.3~33 min were screened by ChemPattern, and peak numbers (1-73) have corresponded with peak numbers in BPI and shown in **Table 3**.

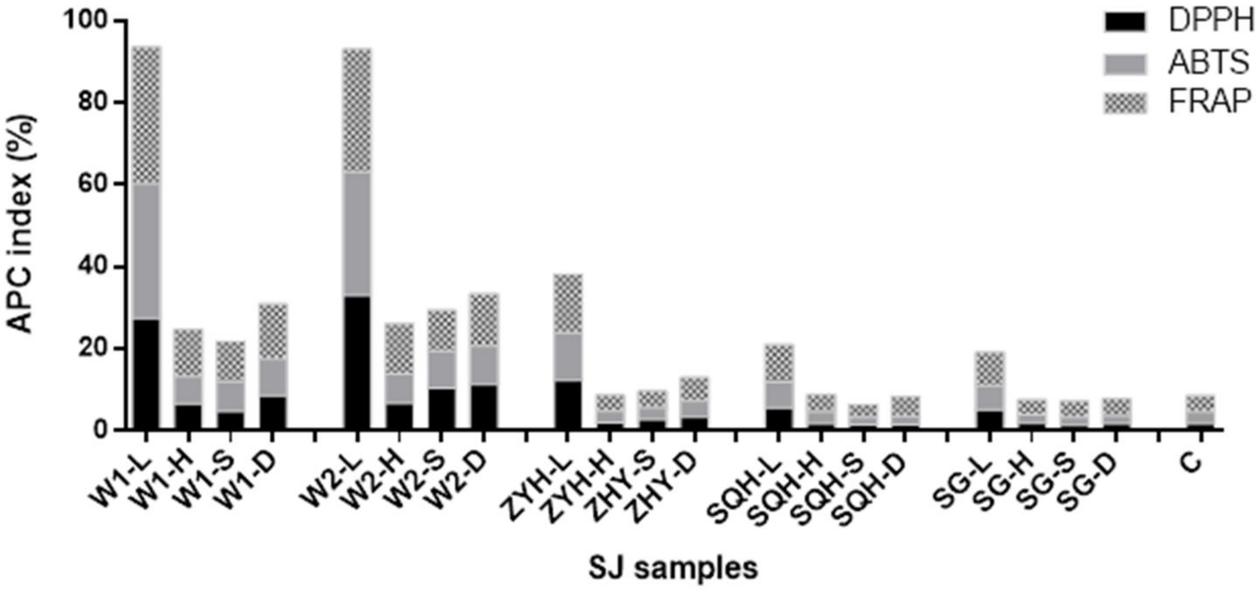

Figure S2. Antioxidant Potency Composite (APC) index of sea buckthorn.

Table S1. The longitudinal diameter (mm) of sea buckthorn berries.

	1	2	3	4	5	6	7	8	9	10	Mean (mm)
W1	5.52	5.39	5.24	5.03	5.62	4.94	6.40	5.78	5.72	5.00	5.46
W2	7.97	7.18	6.87	7.21	7.14	6.51	6.15	7.52	7.12	7.29	7.10
ZYH	8.58	8.68	8.43	9.17	9.13	8.12	8.22	8.58	8.45	9.02	8.64
SQH	10.86	11.27	10.38	12.08	11.57	11.90	11.21	11.93	12.74	12.72	11.67
SG	8.40	10.31	9.11	10.53	9.34	9.07	9.89	9.88	10.21	9.96	9.67

Table S2. (A) Regression equation, LOQ and LOD of the twelve analyzed compounds.

No.	Compounds	Abbreviations	Regression equation	R^2	Linear range (μg/mL)	LOQ (μg/mL)	LOD (μg/mL)
1	Gallic acid	GA	y = 0.3138x − 0.024	0.9996	0.88–112.06	0.2918	0.0875
2	Protocatechuic acid	**PA**	y = 0.3307x + 0.0191	0.9999	0.39–50.40	0.3937	0.1312
3	Rutin	**RU**	y= 0.2872x + 0.4371	0.9998	4.94–632.34	1.2350	0.3088
4	Isoquercitrin	**Q3G**	y = 0.3881x + 0.051	0.9999	1.35–172.46	0.3368	0.1123
5	Isorhamnetin-3-O-neohesperidoside	**I3N**	y = 0.2876x + 0.0713	0.9998	0.89–114.01	0.5938	0.1485
6	Kaempferol-3-O-rutinoside	**K3R**	y = 0.2507x + 0.0544	0.9998	0.94–120.96	0.4725	0.1181
7	Astragalin	**K3G**	y = 0.3641x + 0.1764	0.9997	0.97–124.55	0.3244	0.0811
8	narcissin	**I3R**	y = 0.3108x + 0.5595	0.9998	5.27–674.30	0.4390	0.1646
9	Isorhamnetin-3-O-glucoside	**I3G**	y = 0.4282x + 0.2911	0.9998	2.58–330.54	0.3228	0.1076
10	Quercetin	**QE**	y = 0.633x − 0.1523	0.9999	0.48–30.54	0.4772	0.2386
11	Kaempferol	**KA**	y = 0.6808x − 0.0481	0.9999	0.36–23.23	0.3630	0.0605
12	Isorhamnetin	**IS**	y = 0.398x − 0.0449	0.9998	0.46–29.46	0.4603	0.1534

Table S2. (B) Recovery rates for the seven analyzed components.

Compounds	Original (mg)	Spiked (mg)	Found (mg)	Recovery (%)	Mean (%)	RSD（%）	Compounds	Original (mg)	Spiked (mg)	Found (mg)	Recovery (%)	Mean (%)	RSD（%）
**GA**	0.25	0.25	0.50	102.78	95.78	3.72	**K3G**	0.01	0.01	0.20	101.77	101.98	2.32
	0.22	0.22	0.44	95.62				0.01	0.01	0.20	98.09		
	0.23	0.23	0.45	95.06				0.01	0.01	0.20	101.86		
	0.23	0.23	0.44	93.70				0.01	0.01	0.20	104.19		
	0.23	0.23	0.44	92.86				0.01	0.01	0.20	101.26		
	0.23	0.23	0.44	94.66				0.01	0.01	0.20	104.69		
**PA**	0.00	0.06	0.06	104.09	102.94	2.08	**I3R**	0.54	0.52	1.03	94.11	95.47	3.17
	0.00	0.06	0.06	99.89				0.52	0.52	1.00	93.36		
	0.00	0.06	0.06	100.67				0.51	0.51	1.03	101.33		
	0.00	0.06	0.06	103.42				0.54	0.52	1.03	95.18		
	0.00	0.06	0.06	105.09				0.51	0.51	1.00	95.60		
	0.00	0.06	0.06	104.48				0.53	0.52	1.02	93.22		
**RU**	0.19	0.19	0.38	102.47	99.48	2.79	**I3G**	0.12	0.12	0.23	95.63	97.38	4.22
	0.18	0.18	0.36	94.19				0.12	0.12	0.23	96.36		
	0.18	0.18	0.37	100.51				0.12	0.12	0.23	93.38		
	0.18	0.18	0.37	99.91				0.12	0.12	0.24	99.64		
	0.18	0.18	0.37	99.77				0.12	0.12	0.24	104.60		
	0.19	0.19	0.37	100.01				0.12	0.12	0.23	94.69		
**Q3G**	0.10	0.10	0.21	103.34	100.83	2.73	**QE**	0.00	0.05	0.05	103.31	99.99	3.42
	0.10	0.10	0.20	103.51				0.00	0.05	0.05	95.62		
	0.10	0.10	0.19	96.72				0.00	0.05	0.05	95.74		
	0.10	0.10	0.20	102.02				0.00	0.05	0.05	101.01		
	0.10	0.10	0.19	98.36				0.00	0.05	0.05	101.87		
	0.10	0.10	0.19	101.05				0.00	0.05	0.05	102.40		

**I3N**	0.02	0.02	0.04	99.27	97.59	4.96	**KA**	0.00	0.00	0.01	93.86	93.59	2.24
	0.02	0.02	0.03	94.62				0.00	0.00	0.01	97.09		
	0.02	0.02	0.03	92.90				0.00	0.00	0.01	94.78		
	0.02	0.02	0.03	92.74				0.00	0.00	0.01	91.61		
	0.02	0.02	0.04	102.17				0.00	0.00	0.01	92.13		
	0.02	0.02	0.04	103.85				0.00	0.00	0.01	92.10		
**K3R**	0.04	0.04	0.08	102.41	100.44	4.36	**IS**	0.01	0.01	0.01	100.87		1.48
	0.04	0.04	0.07	97.79				0.01	0.01	0.01	104.81		
	0.04	0.04	0.08	95.68				0.01	0.01	0.01	103.67		
	0.04	0.04	0.08	103.46				0.01	0.01	0.01	102.54		
	0.04	0.04	0.08	96.64				0.01	0.01	0.01	104.69		
	0.04	0.04	0.08	106.67				0.01	0.01	0.01	104.40		

GA (gallic acid), **PA** (protocatechuic acid), **RU** (rutin), **Q3G** (isoquercitrin), **I3N** (isorhamnetin-3-O-neohesperidoside), **K3R** (kaempferol-3-O-rutinoside), **K3G** (astragalin), **I3R** (narcissin), **I3G** (isorhamnetin-3-O-glucoside), **QE** (quercetin), **KA** (kaempferol), **IS** (isorhamnetin).

Table S3. The content of 12 flavonoids and phenolic acids in sea buckthorn berries of different varieties and drying methods.

	**W1 (mg·100 g^{-1})**				**W2 (mg·100 g^{-1})**			
	**L**	**H**	**S**	**D**	**L**	**H**	**S**	**D**
**GA**	49.22 ± 0.39^{a}	24.72 ± 0.12^{b}	52.17 ± 0.08^{a}	53.12 ±1.11^{a}	61.32 ± 0.98^{a}	29.80 ±1.70^{c}	41.57 ± 3.63^{b}	45.92 ± 2.74^{b}
**PA**	0.00 ± 0.00^{c}	3.85 ± 0.05^{ab}	3.75 ± 0.12^{b}	4.20 ± 0.26^{a}	1.42 ± 0.05^{d}	6.26 ± 0.11^{a}	5.52 ± 0.28^{a}	4.67 ± 0.40^{a}
**RU**	35.01 ± 0.24^{a}	24.67 ± 0.07^{c}	29.58 ± 0.51^{b}	28.82 ± 1.55^{b}	24.78 ± 0.37^{a}	16.76 ± 0.16^{c}	19.88 ± 0.81^{b}	20.30 ± 1.00^{b}
**Q3G**	19.13 ± 0.27^{a}	13.70 ± 0.02^{c}	16.24 ± 0.51^{b}	15.88 ± 0.94^{b}	14.75 ± 0.28^{a}	9.33 ± 0.05^{c}	11.72 ± 0.47^{b}	11.77 ± 0.56^{b}
**I3N**	5.94 ± 0.01^{a}	2.94 ± 0.11^{b}	2.98 ± 0.23^{b}	3.05 ± 0.16^{b}	2.43 ± 0.04^{b}	2.36 ± 0.09^{b}	3.20 ± 0.06^{a}	1.41 ± 0.07^{c}
**K3R**	8.28 ± 0.07^{a}	5.24 ± 0.01^{c}	6.72 ± 0.05^{b}	6.27 ± 0.37^{b}	2.35 ± 0.07^{a}	2.18 ± 0.05^{a}	1.57 ± 0.12^{b}	1.52 ± 0.07^{b}
**K3G**	1.07 ± 0.03^{a}	1.26 ± 0.04^{a}	1.10 ± 0.14^{a}	0.71 ± 0.03^{b}	0.00 ± 0.00	0.00 ± 0.00	0.00 ± 0.00	0.00 ± 0.00
**I3R**	101.57 ± 0.59^{a}	72.39 ± 0.07^{c}	81.05 ± 1.63^{b}	79.27 ± 4.72^{b}	122.79 ± 1.89^{a}	84.94 ± 0.81^{c}	107.84 ± 4.21^{b}	106.85 ± 4.99^{b}
**I3G**	24.15 ± 0.29^{a}	15.95 ± 0.10^{d}	17.14 ± 0.44^{c}	18.33 ± 0.65^{b}	23.07 ± 0.50^{a}	15.62 ± 0.13^{c}	19.22 ± 0.69^{b}	19.37 ± 1.18^{b}
**QE**	0.00 ± 0.00	0.00 ± 0.00	0.00 ± 0.00	0.00 ± 0.00	0.00 ± 0.00	0.00 ± 0.00	0.00 ± 0.00	0.00 ± 0.00
**KA**	0.68 ± 0.01^{a}	0.44 ± 0.01^{c}	0.50 ± 0.01^{b}	0.48 ± 0.02b	0.00 ± 0.00	0.00 ± 0.00	0.00 ± 0.00	0.00 ± 0.00
**IS**	1.39 ± 0.04^{a}	1.21 ± 0.02^{b}	1.21 ± 0.06^{b}	1.11 ± 0.01^{c}	0.79 ± 0.06^{b}	1.09 ± 0.09^{b}	2.84 ± 0.55^{a}	0.54 ± 0.02^{c}
SUM	246.45 ± 0.76a	166.32 ± 0.44^{c}	211.75 ± 2.75^{b}	211.24 ± 8.13^{b}	253.71 ± 2.37^{a}	168.34 ± 2.27^{c}	213.37 ± 9.41^{b}	212.35 ± 8.81^{b}

	**ZYH (mg·100 g-1)**				**SQH (mg·100 g^{-1})**			
	**L**	**H**	**S**	**D**	**L**	**H**	**S**	**D**
**GA**	42.61 ± 0.28^{a}	29.10 ± 0.75^{c}	34.87 ± 1.46^{b}	36.49 ± 1.37^{b}	29.22 ± 0.42^{a}	7.55 ± 1.59^{c}	21.13 ± 0.57^{b}	18.71 ± 1.03^{b}
**PA**	0.00 ± 0.00^{d}	14.94 ± 0.30^{a}	6.47 ± 0.05^{b}	3.98 ± 0.14^{c}	0.00 ± 0.00^{d}	23.35 ± 2.08^{a}	15.13 ± 0.14^{b}	10.90 ± 0.10^{c}
**RU**	25.04 ± 0.23^{a}	22.67 ± 0.63^{b}	14.87 ± 0.47^{d}	19.84 ± 0.25^{c}	10.09 ± 0.08^{a}	5.54 ± 0.59^{b}	4.15 ± 0.16^{c}	5.57 ± 0.21^{b}
**Q3G**	23.58 ± 0.09^{a}	19.66 ± 0.53^{b}	14.48 ± 0.44^{d}	17.31 ± 0.68^{c}	11.39 ± 0.08^{a}	5.19 ± 0.58^{c}	6.07 ± 0.45^{bc}	6.40 ± 0.13^{b}
**I3N**	0.73 ± 0.04^{c}	1.30 ± 0.04^{b}	1.94 ± 0.07^{a}	1.84 ± 0.07^{a}	1.02 ± 0.09^{d}	1.89 ± 0.24^{c}	3.10 ± 0.04^{a}	2.44 ± 0.05^{b}
**K3R**	0.00 ± 0.00^{b}	1.07 ± 0.09^{a}	0.95 ± 0.07^{a}	0.00 ± 0.00^{b}	0.00 ± 0.00^{b}	0.00 ± 0.00^{b}	0.63 ± 0.01^{a}	0.00 ± 0.00^{b}
**K3G**	0.00 ± 0.00	0.00 ± 0.00	0.00 ± 0.00	0.00 ± 0.00	0.00 ± 0.00	0.00 ± 0.00	0.00 ± 0.00	0.00 ± 0.00

**I3R**	178.00 ± 1.63^{a}	151.22 ± 4.04^{b}	110.18 ± 2.87^{d}	141.00 ± 2.49^{c}	131.55 ± 1.15^{a}	71.94 ± 6.30^{b}	66.08 ± 1.38^{b}	72.00 ± 0.29^{b}
**I3G**	46.76 ± 0.16^{a}	32.63 ± 0.75^{b}	28.28 ± 0.71^{c}	31.17 ± 0.75^{b}	32.73 ± 0.03^{a}	17.48 ± 1.57^{c}	20.20 ± 0.43^{b}	20.26 ± 0.95^{b}
**QE**	0.78 ± 0.01^{a}	0.72 ± 0.01^{b}	0.69 ± 0.02^{bc}	0.68 ± 0.01^{c}	1.33 ± 0.06^{a}	0.81 ± 0.10^{b}	0.77 ± 0.01^{b}	0.00 ± 0.00^{c}
**KA**	2.22 ± 0.02^{a}	1.78 ± 0.03^{b}	1.47 ± 0.06^{d}	1.58 ± 0.04^{c}	3.16 ± 0.01^{a}	1.79 ± 0.16^{b}	2.38 ± 0.04^{b}	2.30 ± 0.06^{b}
**IS**	1.84 ± 0.04^{b}	3.21 ± 0.60^{a}	1.56 ± 0.01^{b}	1.56 ± 0.03^{b}	7.37 ± 0.29^{b}	5.88 ± 0.92^{bc}	11.96 ± 0.13^{a}	5.04 ± 0.65^{c}
SUM	321.56 ± 1.85^{a}	278.28 ± 6.05^{b}	215.77 ± 5.74^{d}	255.44 ± 4.15^{c}	227.86 ± 0.49^{a}	141.32 ± 14.05^{b}	151.98 ± 2.39^{b}	144.90 ± 0.85^{b}

	**SG (mg·100 g^{-1})**				**C (mg·100 g^{-1})**
	**L**	**H**	**S**	**D**	**L**
**GA**	27.41 ± 0.52^{a}	16.51 ± 0.54^{c}	17.63 ± 1.63^{c}	21.92 ± 1.09^{b}	19.94 ± 1.05
**PA**	0.00 ± 0.00^{d}	36.85 ± 0.06^{a}	13.05 ± 0.37^{b}	13.52 ± 0.26^{bc}	14.58 ± 0.07
**RU**	39.23 ± 0.18^{a}	31.26 ± 0.38^{b}	18.32 ± 0.61^{d}	27.27 ± 0.63^{c}	24.16 ± 0.93
**Q3G**	32.34 ± 0.00^{a}	24.84 ± 1.01^{b}	18.03 ± 0.46^{c}	23.48 ± 0.19^{b}	16.90 ± 0.77
**I3N**	3.29 ± 0.01^{a}	1.53 ± 0.06^{d}	1.77 ± 0.06^{c}	2.41 ± 0.08^{b}	1.36 ± 0.11
**K3R**	3.09 ± 0.09^{a}	2.49 ± 0.10^{b}	2.46 ± 0.16^{b}	1.83 ± 0.03^{c}	1.69 ± 0.07
**K3G**	0.00 ± 0.00^{b}	0.00 ± 0.00^{b}	0.19 ± 0.02^{a}	0.00 ± 0.00^{b}	0.00 ± 0.00
**I3R**	377.25 ± 2.00^{a}	286.42 ± 0.84^{b}	216.79 ± 6.06^{d}	260.24 ± 5.29^{c}	158.04 ± 3.74
**I3G**	83.40 ± 0.23^{a}	62.68 ± 0.25^{b}	53.27 ± 1.26^{d}	58.22 ± 0.85^{c}	30.34 ± 0.97
**QE**	0.00 ± 0.00^{b}	0.85 ± 0.05^{a}	0.00 ± 0.00^{b}	0.00 ± 0.00^{b}	0.50 ± 0.01
**KA**	5.05 ± 0.01^{a}	3.27 ± 0.02^{b}	3.04 ± 0.07^{c}	3.36 ± 0.06^{b}	1.83 ± 0.03
**IS**	5.32 ± 0.11^{c}	13.59 ± 1.16^{a}	9.01 ± 0.76^{b}	6.69 ± 0.36^{c}	3.03 ± 0.12
SUM	576.37 ± 2.48^{a}	480.29 ± 3.81^{b}	353.57 ± 9.84^{d}	419.44 ± 6.66^{c}	272.35 ± 7.78

Values were expressed as the mean ± standard deviation, n=3. Significant differences between different drying methods of each variety ($p < 0.05$) are marked a-d. **GA** (gallic acid), **PA** (protocatechuic acid), **RU** (rutin), **Q3G** (isoquercitrin), **I3N** (isorhamnetin-3-O-neohesperidoside), **K3R** (kaempferol-3-O-rutinoside), **K3G** (astragalin), **I3R** (narcissin), **I3G** (isorhamnetin-3-O-glucoside), **QE** (quercetin), **KA** (kaempferol), **IS** (isorhamnetin).
